# Work stress, work-family conflict, and psychological distress among resort employees: a JD-R model and spillover theory perspectives

**DOI:** 10.3389/fpsyg.2024.1326181

**Published:** 2024-02-14

**Authors:** Ahmed Hassan Abdou, Maha Abdul-Moniem Mohammed El-Amin, Elham Farouq Ali Mohammed, Hanem Mostafa Mohamed Alboray, Aza Mohamed Sediek Refai, Muhanna Yousef Almakhayitah, Abdullah Saleh Mohammed Albohnayh, Abdulaziz Mohammed Alismail, Mazen Omar Almulla, Jawharah Saleh Alsaqer, Maha Hassanein Mahmoud, Adel Ibrahim Abdullah Elshazly, Sahar Farouk Abdelgaed Allam

**Affiliations:** ^1^Social Studies Department, College of Arts, King Faisal University, Al-Ahsa, Saudi Arabia; ^2^Hotel Studies Department, Faculty of Tourism and Hotels, Mansoura University, Mansoura, Egypt; ^3^Department of Education and Psychology, College of Education, King Faisal University, Al-Ahsa, Saudi Arabia; ^4^Education Fundamentals Department, Applied College, King Faisal University, Al-Ahsa, Saudi Arabia; ^5^Mental Health Department, Faculty of Education, Suez Canal University, Ismailia, Egypt; ^6^Department of Psychology, Faculty of Arts, Helwan University, Cairo, Egypt; ^7^Department of Geography, College of Arts, King Faisal University, Al-Ahsa, Saudi Arabia; ^8^Department of Geography, College of Arts, Mansoura University, Mansoura, Egypt; ^9^Department of Curricula and Teaching Methods, College of Education, King Faisal University, Al-Ahsa, Saudi Arabia; ^10^Department of Psychology, Faculty of Women, Ain Shams University, Cairo, Egypt

**Keywords:** resort industry, psychological wellbeing, work-related stressors, psychological strains, family-life conflict, WFC

## Abstract

The hospitality industry is well-known for its challenging and high-pressure work settings. In this context, employees commonly face a multitude of stressors originating from their roles and job responsibilities, which can significantly impact their psychological wellbeing. Hence, based on the job demands-resources (JD-R) model and the spillover theory, this study aims to empirically explore the direct and indirect effect of work stress (assessed by role overload, ambiguity, and conflict) on psychological distress among frontline employees in 3- and 4-star Egyptian resorts while considering the mediating influence of work-family conflict (WFC). Four hypotheses were put to the test through the application of the PLS-SEM 4.0 version (4.0.9.9). Based on the findings from 563 frontline employees who participated in this research, the study supports the four hypotheses affirming that work-related stressors significantly contributed to employees' psychological distress. Further, the findings highlighted that these stressors significantly spill over into employees' family lives, generating conflicts between work and family roles. In addition, the results emphasized the significance of WFC as a contributing factor to employees' psychological distress. Finally, the study concluded that WFC partially mediates the link between work stress and employees' psychological distress. Based on these findings, some theoretical and practical implications for hospitality scholars, resort management, and policymakers were suggested to enhance the employees' wellbeing and mitigate psychological distress in this vital sector.

## 1 Introduction

The hospitality industry, while known for its vibrant nature and focus on providing exceptional service to its guests, faces a unique challenge in the form of work-related stress among its employees (Pizam and Shani, 2009; Walker, 2021). The hospitality industry's constant operation, often 24/7, requires employees to work irregular hours, including evenings, weekends, and holidays (O'Neill and Xiao, 2010). This disrupts work-life balance, making it difficult to fulfill personal and family obligations (Zhao and Ghiselli, 2016; Yousaf et al., 2020; Abdou et al., 2022). Furthermore, the industry's emphasis on customer satisfaction puts immense pressure on employees to consistently deliver exceptional service (Kusluvan, 2003). Frontline staff, like front desk personnel and waiters, directly interact with customers, managing their diverse requests and expectations (Wang et al., 2021; Pradhan, 2022). This customer-centric environment can be emotionally demanding, requiring employees to constantly strive to meet and exceed customer demands. Unchecked work stress from these factors can have significant consequences, negatively impacting employees' emotional and psychological wellbeing (Foster et al., 2020; Shen and Slater, 2021).

Psychological distress is a multifaceted and complex phenomenon encompassing individuals' various emotional and mental health challenges (Ridner, 2004). It represents a state of emotional suffering and discomfort that can manifest in various forms, such as anxiety, depression, and a general sense of unease (Ridner, 2004; Winefield et al., 2012). Numerous factors, including work-related stressors, and work-family conflict can trigger the experience of psychological distress (Janzen et al., 2007; Huang et al., 2021). It is a pervasive issue that affects people of all ages, backgrounds, and walks of life, transcending cultural, geographical, and socioeconomic boundaries. Psychological distress can have profound and lasting consequences on an individual's mental, emotional, and physical health and overall quality of life (Pomaki et al., 2004; Kilpatrick et al., 2013; Martins and Lopes, 2013). In this context, it is crucial to recognize and address psychological distress and its antecedents within the workplace to promote employees' wellbeing and prevent the escalation of symptoms.

In today's fast-paced and demanding work environments, achieving a harmonious balance between work-related obligations and family responsibilities has become increasingly challenging (Byron, 2005; Michel et al., 2011). Work-family conflict (WFC) arises when the demands, pressures, or commitments associated with one domain (i.e., work) interfere with an individual's ability to fulfill responsibilities in the other domain (i.e., family) (Greenhaus and Beutell, 1985). This conflict can manifest in various forms: time-based, strain-based, and behavior-based (Frone et al., 1992). The concept of WFC has gained prominence in the context of work stress and its repercussions on employees' wellbeing (Al-Jubari et al., 2022; Huo and Jiang, 2023). It serves as a pivotal mechanism through which stressors in the workplace can impact individuals' personal lives and vice versa. The bidirectional nature of WFC underscores its significance in understanding the interplay between work stress and psychological distress (Haines III et al., 2008; Shimazu et al., 2010; du Prel and Peter, 2015; Oshio et al., 2017).

While there is a considerable body of research on work stress, psychological distress, and WFC in various occupational settings, there is a noticeable gap in the context of resort employees in developing countries such as Egypt (Abdou et al., 2022). As suggested by Abdou et al. (2022), the Egyptian hospitality industry presents unique challenges, with many resorts in remote areas with limited local labor and seasonal fluctuations in demand. These factors contribute to irregular work patterns and challenges in maintaining long-term employment (Soliman et al., 2023). Additionally, many resort employees come from distant regions and live at the resort for extended periods with high instances of homelessness, food insecurity, ever-shifting work schedules, extra-long commutes, low wages, and separation from their primary residences (Dreier et al., 2018). These unique circumstances can significantly impact their experience of WFC and psychological distress. Moreover, while some studies have explored the direct relationship between work stress and psychological distress, there may be a gap in comprehensively examining how work stress affects employees' psychological distress. Research that delves into mediating mechanisms, such as WFC, may be limited in the context of resort workers' experiences. Finally, our research is a direct response to the call by Abdou et al. (2022) for further investigations into the mediating role of WFC among various variables within the hospitality industry.

To address this gap, this study aims to investigate the impact of work-related stressors (role overload, ambiguity, conflict) on psychological distress among frontline employees in Egyptian resorts, considering the mediating role of WFC. It draws on the (Bakker and Demerouti, 2017) and Spillover theory (Staines, 1980) to explore the following research questions: (1) What is the nature and extent of work stress experienced by frontline employees in the resort industry? (2) To what extent does work stress affect WFC and psychological distress among resort employees? and (3) To what extent does WFC mediate the relationship between work stress and psychological distress among resort employees?

The Job Demands-Resources (JD-R) model elucidates work experiences, incorporating demands and resources impacting wellbeing and behaviors. Resources, like supportive colleagues, clear job descriptions, and training, foster personal growth and wellbeing (Llorens et al., 2006; Bauer et al., 2014). Job demands, requiring sustained effort, encompass role overload, conflict, and ambiguity, straining employees and yielding adverse outcomes (Rizzo et al., 1970; Hecht, 2001; Creary and Gordon, 2016). When employees are consistently exposed to high levels of job demands, they are more likely to experience a depletion of their resources, leading to feelings of strain and eventual psychological distress. In this study, assessing the resources component of Bakker and Demerouti's model fell outside the study's intended scope.

In addition, the study integrates the spillover theory with the JD-R model, examining how stressors in work spill over into family life. Spillover theory recognizes life domains' interconnectedness, where events in one domain affect another (Staines, 1980). Work-family spillover specifically explores how work experiences or stressors impact family life (Grzywacz et al., 2002; Wayne et al., 2017). For instance, a stressful workday can lead to negative emotions carried home, potentially affecting family interactions (Sirgy et al., 2020). Accumulated stress and negative emotions in both work and family domains contribute to psychological distress, manifested as anxiety, depression, burnout, or reduced overall wellbeing.

By addressing these dynamics, we seek to provide valuable insights for resort management and policymakers to enhance the wellbeing of employees in this vital sector. The focus is on understanding the potential negative effects of work-related stressors on employee wellbeing, providing a basis for developing strategies to enhance mental health. Additionally, the study investigates the role of WFC as a crucial mediator, offering insights into the specific mechanisms through which work stress influences psychological distress. This knowledge can inform targeted interventions to reduce WFC and alleviate the adverse consequences of work stress. Furthermore, the study may contribute to the existing literature by exploring the applicability of the JD-R model and spillover theory in the hospitality industry, providing a more comprehensive understanding of how work-related stressors impact employees' family lives and contribute to psychological distress. The integrated theoretical frameworks offer a deeper exploration of the underlying mechanisms in this specific industry context.

## 2 Theoretical background and hypothesis development

### 2.1 The impact of work stress on employees' psychological distress

Work stress (WS) is a pervasive and increasingly prevalent phenomenon in today's fast-paced and competitive work environments. It is recognized as a multifaceted issue affecting individuals, organizations, and society (Hon and Chan, 2013; Thorsteinsson et al., 2014; Yousaf et al., 2020). Work stress, or occupational stress, can be described as the adverse reaction individuals experience when they perceive a discrepancy between their work demands and their ability to cope effectively (Lo and Lamm, 2005; Yousaf et al., 2020). It is often characterized by feelings of pressure, tension, and emotional strain resulting from various factors within the work context (Murray-Gibbons and Gibbons, 2007; Hwang et al., 2014). Work stress is not a one-size-fits-all concept; instead, it is a complex and dynamic phenomenon influenced by a wide range of factors. These factors encompass both the external aspects of the work environment and individual characteristics, creating a diverse landscape of stressors and responses (Rao and Goel, 2018; Khuong and Linh, 2020).

In the hospitality sector, three significant stressors—role conflict, ambiguity, and overload—contribute to work-related stress among employees (Karatepe, 2013; Khalil et al., 2020; Unguren and Arslan, 2021; Elshaer et al., 2022; Salama et al., 2022). Role conflict arises from conflicting demands in handling diverse guest needs, making it challenging to provide consistent service (Rizzo et al., 1970; Peterson et al., 1995). Role ambiguity occurs when employees are uncertain about job expectations, leading to confusion, stress, and decreased job satisfaction (Schmidt et al., 2014; Inoue et al., 2018). Role overload is common, with employees expected to fulfill numerous responsibilities within limited timeframes due to factors like high guest expectations and fluctuating customer volumes. This results in multitasking, simultaneous duty balancing, and extended working hours to meet role demands in the hospitality industry (Lin and Ling, 2018; Elshaer et al., 2024).

As one of its consequences, many researchers have emphasized the notion that work stress serves as a critical determinant of employees' psychological distress. Employees' psychological distress refers to the emotional and mental strain experienced by individuals in a workplace environment (Fordjour et al., 2020; Chan et al., 2021). It encompasses feelings of anxiety, depression, frustration, and overall emotional discomfort resulting from various work-related factors, such as excessive workload, interpersonal conflicts at work, and job insecurity (Ridner, 2004; Winefield et al., 2012; Wu et al., 2012). Existing literature strongly supports the significant positive relationship between work stress and employees' psychological distress (Iwata et al., 1992; Revicki and Gershon, 1996; Wang and Wang, 2019; Li et al., 2020). For instance, in the context of Chinese physicians working in general hospitals, Wang and Wang (2019) have found a significant positive relationship between work stress and employees' psychological distress, indicating that as work-related stressors increase, the likelihood of experiencing psychological distress also increases. Moreover, in the Chinese nurse setting, Xiao et al. (2022) demonstrated that role stress, including role conflict, ambiguity, and overload, has been linked to various forms of psychological distress, including symptoms of anxiety, depression, burnout, and stress. Similarly, role conflict often leads to increased psychological distress among employees. When employees are torn between competing demands from their roles, they experience higher levels of stress, which can manifest as psychological and emotional symptoms of distress as individuals struggle to balance conflicting demands (Alyamy and Sau Cheong, 2020; Pretorius and Padmanabhanunni, 2022). Finally, based on the JD-R model, individuals working in high-strain jobs characterized by high job demands and low job resources are more likely to experience adverse health effects, including psychological distress. Accordingly, it could be postulated that.

*H1: Work-related stressors (including role conflict, ambiguity, and overload) have a significant effect on employees' psychological distress*.

### 2.2 The impact of work stress on WFC

WFC was defined as “A form of inter-role conflict in which the role pressures from the work and family domains are mutually incompatible in some respect” (Greenhaus and Beutell, 1985, p. 77). WFC is exhibited in three forms, each presenting its unique set of challenges. Time-based conflict arises when the time allocated to one's professional commitments encroaches upon the time needed for family responsibilities, leaving individuals torn between competing priorities. Behavior-based conflict occurs when the behaviors and attitudes expected in one role are incongruent with those expected in the other, leading to role-related stress and identity clashes. Strain-based conflict arises when stress, exhaustion, or emotional strain experienced in one domain spills over into the other, impairing one's ability to function effectively in both spheres (Greenhaus and Beutell, 1985; Mihelic and Tekavcic, 2014; Allen et al., 2020).

Several studies have examined the relationship between work stress and WFC to understand how work-related stressors can spill over into individuals' family lives (Michel et al., 2011). They emphasized that work stressors can spill over into an individual's family life, making it challenging to detach from work-related concerns. For instance, earlier studies (i.e., Michel et al., 2011; Mohamad et al., 2016; Rubel et al., 2017; Mohd Isa et al., 2018; Suhartini et al., 2023) suggested that higher levels of role overload, conflict, and ambiguity are associated with increased WFC. When employees are overwhelmed with work-related responsibilities, they may need help to engage fully with their family members, participate in family activities, or fulfill their caregiving roles (Dodanwala et al., 2022). In another empirical study, researchers indicated a notable increase in WFC when employees reported heightened role conflict and ambiguity levels within their organization. More specifically, they found that when employees are unsure about their roles at work, it can result in stress and anxiety, which can spill over into their family lives (Mohamad et al., 2016). Role ambiguity can lead to WFC as individuals struggle to manage their responsibilities effectively, often bringing work-related stressors home. In a similar vein, role conflict within the workplace can have a significant impact on WFCs experienced by employees (Farika et al., 2021). Employees dealing with conflicting expectations and demands at work may experience spillover effects into their family life, resulting in heightened stress levels. Hence, based on the spillover theory, it could be hypothesized that.

*H2: Work-related stressors (including role conflict, ambiguity, and overload) have a significant contribution to the increase of WFC*.

### 2.3 The impact of WFC on employees' psychological distress

Numerous scholars have addressed the relationship between WFC and employees' psychological distress across various industry sectors (Janzen et al., 2007; Kafetsios, 2007; Jacobsen et al., 2014; Aazami et al., 2015; Bilodeau et al., 2020). They highly emphasized the notion that WFC has a substantial impact on employees' psychological distress. Specifically, when individuals experience conflict between their work and family roles, it can lead to various negative psychological outcomes. For instance, some scholars revealed that prolonged exposure to WFC can lead to burnout, characterized by emotional exhaustion, cognitive weariness, depersonalization, and reduced personal accomplishment (Karatepe et al., 2010; Wang et al., 2012; Barriga Medina et al., 2021). Further, Panatik et al. (2012) suggested a positive association between WFC and psychological distress. When employees experience conflict between their work and family roles, it can lead to increased stress and strain, which in turn can contribute to psychological distress. Another study found that WFC can be a significant source of stress for employees (Poms et al., 2016). The demands and expectations of balancing work and family responsibilities can create chronic stress. This stress can manifest as feelings of overwhelm, anxiety, and tension, contributing to psychological distress (Poms et al., 2016; Rubab, 2017). Additionally, during the COVID-19 pandemic, WFC has been significantly associated with an increased risk of mental health issues, including symptoms of depression, insomnia, and anxiety. These mental health challenges indicate psychological distress (Antino et al., 2022). As a result, it could be hypothesized that.

*H3: Perceived WFC has a significant positive effect on employees' psychological distress*.

### 2.4 The mediating role of WFC in the link between work stress and employees' psychological distress

While numerous studies have undoubtedly highlighted the substantial positive influence of work stress on the emergence of WFC and concurrent psychological distress among employees (i.e., Ryan et al., 2015; Fordjour et al., 2020; O'Neill and Follmer, 2020; Chan et al., 2021), and have underscored the noteworthy role of WFC in amplifying employees' psychological distress (i.e., Kafetsios, 2007; Jacobsen et al., 2014; Bilodeau et al., 2020), it is apparent that a significant dearth of research exists concerning the comprehensive exploration of the intermediating role played by WFC in the link between work-related stressors (including role conflict, ambiguity, and overload) and psychological distress experienced by employees particularly, in the resort industry context. In a non-hospitality context, Oshio et al. (2017) conducted an extensive investigation into the intervening role of WFC within the nexus between work-related stressors and employees' psychological distress. The outcomes of this study affirmed that WFC played a substantial mediating role in the link between job-related stressors and the psychological distress experienced by employees. Further, another empirical study by Haines III et al. (2008) suggested that work-family interference partially mediated the association between depression and shiftwork. In the same vein, du Prel and Peter (2015) observed that WFC significantly partially mediates the nexus between work stress and depressive symptoms. Moreover, research involving 196 working parents with preschool children in Japan demonstrated that the impact of job demands, such as work overload and emotional demands, on employees' psychological distress was partially mediated by WFC (Shimazu et al., 2010). Hence, building upon these previous findings and through the framework of spillover theory, one could posit the following hypothesis.

*H4: WFC has a significant partial mediating effect on the association between work-related stressors and employees' psychological distress*.

**Figure 1:**
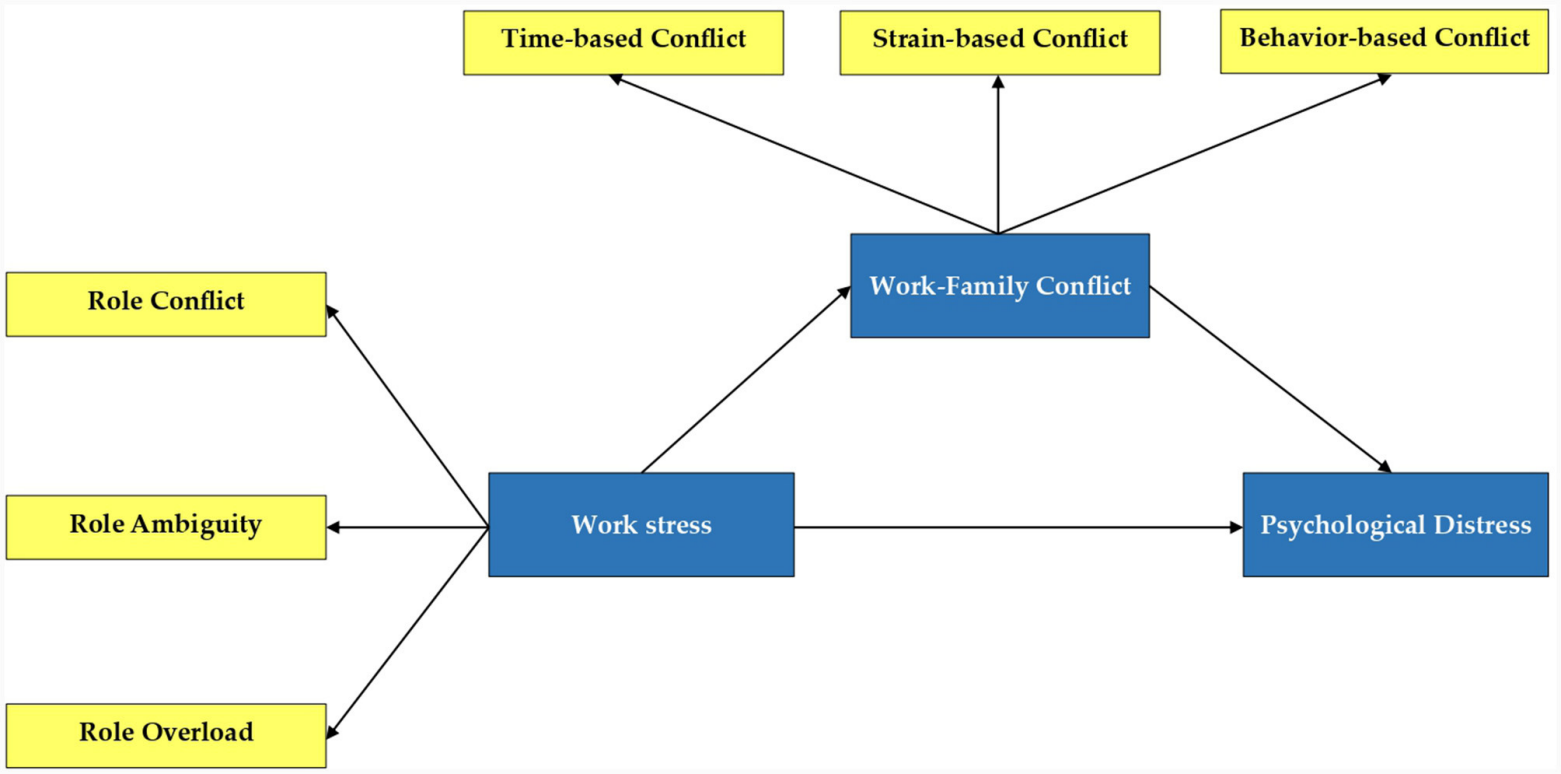
The study's theoretical model. This theoretical model presents the relationship between work stress (independent variable), work-family conflict (mediating variable), and employees' psychological distress (dependent variable).

Figure 1 depicts the study's theoretical framework.

## 3 Materials and methods

### 3.1 Measures and instrument development

As previously stated, this study is designed to empirically investigate the impact of work-related stressors (assessed by role overload, ambiguity, and conflict) on psychological distress among frontline employees in three- and four-star Egyptian resorts while considering the mediating influence of WFC. The primary data collection method employed for this research is online questionnaires. The choice of online questionnaires for data collection offers several advantages that align with modern research's requirements and objectives, including convenience, efficiency, and accessibility.

The questionnaire development involved a literature review to identify scales and items related to work-related stressors, psychological distress, and work-family conflict. A comprehensive pool of potential items for each construct was generated based on the findings. The questionnaire comprised four sections, each serving a specific purpose. Section 1 (demographic data) collected basic demographic information from the participants (i.e., gender, age….etc). Section 2 (work stress) includes role overload, role ambiguity, and role conflict. Based on Peterson et al. (1995), a 13-item scale was utilized, including three items for measuring role conflict, five for role ambiguity, and five for role overload. An example item is “There is a need to reduce some parts of my role.” Section 3 (WFC) measured participants' perceptions of work-family conflict. A 9-item scale, that includes three dimensions each comprising three items, adapted by Abdou et al. (2022) was employed to assess this construct. An illustrative item from these dimensions is “Working in resorts keeps employees from their family activities more than it should be.” Section 4 (psychological distress) aimed to explore participants' psychological distress using the well-established 10-item Kessler psychological distress scale (K10) based on the study by Andrews and Slade (2001). A sample item from this section is “In the past 30 days, how often did you feel depressed?” Responses to work stress and WFC queries were collected on a five-point Likert scale, with one corresponding to “strongly disagree” and five representing “strongly agree.” Meanwhile, the response rate regarding psychological distress was calculated using a five-point Likert scale where one means “none of the time” while five means “all of the time.” Strong internal consistency was observed for the work stress (α = 0.880), work-family conflict (α = 0.897), and psychological distress (α = 0.866) scales.

The survey initially originated in English and was subsequently translated into the native Arabic language of the participants. To ensure linguistic accuracy and consistency, the questionnaire was then reverse-translated from Arabic back to English. This rigorous process was undertaken to confirm that the translated version faithfully retained the intended meanings of the original questions. In addition to the translation process, the questionnaire underwent a comprehensive face validity assessment. Four hospitality human resource management experts were enlisted to evaluate the questionnaire's content critically. Their invaluable feedback and insights were instrumental in refining the questionnaire, ensuring it effectively measured the intended constructs.

Furthermore, a pilot study was conducted involving a separate sample of 30 employees from resorts who were not included in the primary study sample. This pilot study served the purpose of assessing the questionnaire's feasibility. Specifically, it aimed to determine whether the questionnaire was easily comprehensible, appropriate for the context and whether the questions were unambiguous, clearly articulated, and consistently presented. Based on the feedback and comments from the hospitality experts and pilot study participants, necessary modifications were implemented. This included refinements in the wording of certain statements and adjustments to the sequence of questions, all aimed at enhancing the overall clarity and effectiveness of the questionnaire.

### 3.2 Sampling and data collecting

The focal point of this research was frontline employees employed in three- and four-star resorts located in Egypt. Before collecting data from this specific group of participants, a list of three- and four-star resorts in Egyptian destinations, particularly in Hurghada and Sharm El-Shiekh, where most resorts are situated, was established (Abdou et al., 2022). This study mainly focused on frontline employees because they often juggle diverse tasks and deal with demanding guest interactions, which can contribute to work stress and spill over into family life. Given the busy and dynamic nature of resort operations, making it challenging to access participants using random or probability sampling methods, convenience sampling was employed. Convenience sampling was chosen due to its practicality and feasibility in accessing the specific participants within the hospitality industry. Furthermore, it also allowed the researchers to collect data efficiently without excessive time and resource constraints. A research invitation explaining the study's objective was sent to human resource managers seeking authorization for data collection. Only eight resorts (three 3-star and five 4-star resorts) agreed to participate and cooperated with the research team.

Human resource managers were provided with a hyperlink to access the survey form and were requested to distribute it among frontline employees for their responses. A welcome message, along with a concise explanation of the study's objectives, was incorporated. Participants were explicitly informed that participation was voluntary, and they were reminded to review and confirm their responses before the final submission of the survey. Over the data collection period spanning about 2 months (from May to July 2023), a total of 563 survey forms were gathered and subjected to statistical analysis.

The study adheres to the ethical principles and guidelines outlined in the Declaration of Helsinki. All participants provided voluntary and informed consent before participating in the study. They were fully informed about the study's purpose, and procedures. The consent form explicitly stated their right to withdraw from the study at any point without facing any negative consequences. Participants' privacy and confidentiality were protected. Researchers ensured participants that their personal information would remain confidential, and data would be anonymized whenever possible. The research protocol was thoroughly reviewed and approved by the ethical committee of King Faisal University's scientific research deanship. The approval was granted under project number 4605, and the official date of approval was 1 May 2023.

Following Nunnally and Bernstein's (1994) guidance, the sample size for this study was determined while considering the number of items under examination. They recommended an appropriate ratio of 1:10 (item to sample). For instance, with 32 items in this study, a sample size of 320 respondents was considered suitable. In alignment with this recommendation, a sample size of 563 participants was employed in the current study, which is considered sufficient. This choice also aligns with the recommendation by Hair et al. (2019), indicating that a minimum sample size of 155 is required for PLS-SEM when expecting minimum path coefficients (*P*_min_) to range between 0.11 and 0.20 at a significance level of 0.05. Furthermore, the selected sample size adheres to Boomsma's (1982) advice, suggesting that a minimum of 200 samples is suitable for structural equation modeling.

### 3.3 Data analysis

Data analysis for this study was performed with SPSS 25 and SmartPLS 4 version 4.0.9.9 software. Frequencies and percentages were employed to summarize and present the demographic characteristics of the study participants. Further, we employed PLS-SEM along with bootstrapping techniques to examine reliability, validity, as well as multicollinearity and test the hypotheses derived from our research questions.

## 4 Results

### 4.1 Respondents' demographic characteristics

As previously mentioned, the study gathered a total of 563 valid responses. Regarding their gender, a significant majority, comprising 74.8% of the respondents, were males, while the remaining 25.2% were females. Concerning age, 57.6% fell within the 20–30-year age group, followed by the 31–40-year age group, which accounted for 36.2%. The older age group, spanning 40–50 years, constituted the smallest proportion at 6.2%. In terms of educational qualifications, 62.5% of the participants held a university degree, 34.8% possessed a high school degree, and 2.7% held a postgraduate degree. Following their marital status, more than two-thirds (68.4%) were married, 25.8% were single, and the others (i.e., divorced or widowers) represent 5.8%. When considering their departments within the resorts, the food and beverage department had the highest representation at 47.4%, followed by the housekeeping and front office departments at 31.1 and 21.5% respectively. Regarding their work experience in the investigated resorts, 40% had worked for <3 years, 35.7% for 3–5 years, and 24.3% for more than 5 years (see Table 1).

**Table 1:** Demographics characteristics of participants.

**Baseline characteristics**	**No**.	**%**
**Gender**	
Male	421	74.8
Female	142	25.2
**Age**	
20–30 years	324	57.6
31–40 years	204	36.2
40–50 years	35	6.2
**Educational qualification**	
High school	196	34.8
University degree	352	62.5
Postgraduate degree	15	2.7
**Marital status**	
Single	145	25.8
Married	385	68.4
Others (i.e., divorced/widowers)	33	5.8
**Current department**	
Food and beverage	267	47.4
Housekeeping	175	31.1
Front office	121	21.5
**Work experience in the current resort**	
Less than 3 years	225	40
From 3–5 years	201	35.7
More than 5 years	137	24.3

N = 563.

### 4.2 Common method bias

To address the potential issue of common method bias (CMB) in the data collected through an online survey, several measures were implemented. Firstly, anonymity and confidentiality were ensured to reduce the possibility of common method bias (Nancarrow et al., 2001). All information and responses provided by research participants were treated as confidential and anonymous and were solely used for the purposes of the study. Guaranteeing anonymity minimizes the likelihood of response bias (Randall and Fernandes, 1991). Participants were also kindly requested to answer all questions honestly, reducing response bias (Phillips and Clancy, 1972). Furthermore, Harman's single-factor test was conducted to assess the presence of CMB. Through exploratory factor analysis, it was found that one factor explained 38.9% of the variance. If one factor explains more than 50% of the variance, CMB may be a concern. Hence, CMB did not pose a significant issue (Podsakoff et al., 2003).

### 4.3 Assessment of measurement scale

After the data collection phase, an evaluation of the psychometric properties, encompassing the examination of reliability, convergent validity, and discriminant validity of the scale items, was conducted using the PLS-SEM algorithm. The results presented in Table 2 revealed positive psychometric properties. More specifically, the values of Cronbach's α coefficients and composite reliability (CR) values for all latent constructs ranged from 0.866 to 0.897 and 0.934 to 0.975, respectively, surpassing the recommended threshold of 0.70 as suggested by Hair et al. (2019) and implying excellent internal consistency reliability. For evaluating construct validity, we employed measures of convergent and discriminant validity. Convergent validity mandates a factor loading of at least 0.70 and an AVE >0.50 (Hair et al., 2019). In our analysis, all the study items exhibited factor loadings exceeding 0.70, and the AVE for each construct ranged from 0.590 to 0.813, well above the 0.50 threshold, signifying the attainment of convergent validity.

**Table 2:** Constructs' validity and reliability measures.

**Construct**	**Item**	**Outer loading**	**α^1^**	**CR^2^**	**AVE^3^**
Work stress			0.880	0.967	0.697
Role conflict	WS1	0.779^***^	0.749	0.815	0.596
	WS2	0.810^***^			
	WS3	0.724^***^			
Role ambiguity	WS4	0.907^***^	0.899	0.949	0.789
	WS5	0.821^***^			
	WS6	0.835^***^			
	WS7	0.910^***^			
	WS8	0.962^***^			
Role overload	WS9	0.796^***^	0.869	0.909	0.666
	WS10	0.823^***^			
	WS11	0.870^***^			
	WS12	0.811^***^			
	WS13	0.779^***^			
Work-family conflict			0.897	0.975	0.813
Time-based conflict	WFC1	0.885^***^	0.872	0.923	0.801
	WFC2	0.910^***^			
	WFC3	0.889^***^			
Strain-based conflict	WFC4	0.892^***^	0.817	0.908	0.767
	WFC5	0.884^***^			
	WFC6	0.851^***^			
Behavior-based conflict	WFC7	0.905^***^	0.861	0.953	0.873
	WFC8	0.950^***^			
	WFC9	0.947^***^			
Psychological distress	PSY1	0.781^***^	0.866	0.934	0.590
	PSY2	0.856^***^			
	PSY3	0.822^***^			
	PSY4	0.727^***^			
	PSY5	0.705^***^			
	PSY6	0.755^***^			
	PSY7	0.701^***^			
	PSY8	0.828^***^			
	PSY9	0.738^***^			
	PSY10	0.750^***^			

WS, work stress; WFC, work-family conflict; PSY, psychological distress; α^1^, Cronbach's alpha; CR^2^, composite reliability; AVE^3^, average variance extracted.

^***^p < 0.001.

Regarding discriminant validity, two measures were conducted: the Fornell-Larcker criterion and the HTMT ratio. Fornell-Larcker criterion necessitates that the square root of the AVE for each construct should surpass its correlation with other constructs. The data presented in Table 3 demonstrated that the square root of the AVE for all constructs exceeded their correlations with other constructs. In addition, we examined the HTMT ratios, as presented in Table 4, in accordance with the established threshold of 0.85, as advised by Henseler et al. (2015). Significantly, all HTMT ratios in this investigation were observed to be lower than the predefined threshold. This outcome further reinforces the evidence for the presence of discriminant validity among the study constructs.

**Table 3:** Discriminant validity based on fornell–larcker criterion.

**Construct**	**1**	**2**	**3**
1. Psychological distress	**0.768**		
2. Work stress	0.763^a***^	**0.835**	
3. WFC	0.740^a***^	0.475^a***^	**0.902**

The bold diagonal values represent the square root of AVE.

^a^Latent variables' correlation.

^***^p < 0.001.

**Table 4:** Discriminant validity via HTMT.

**Construct**	**1**	**2**	**3**
1. Psychological distress			
2. Work stress	0.835		
3. WFC	0.821	0.538	

All HTMT scores are <0.85.

### 4.4 Multicollinearity statistics

In adherence to the counsel of Hair et al. (2019), this research employed VIF values to evaluate the presence of multicollinearity within the model. The guideline suggests that a VIF score surpassing three is indicative of potential multicollinearity concerns. As indicated by the data in Table 5, all examined constructs in this study exhibit VIF values considerably below the stipulated threshold of three. This outcome proves the absence of notable multicollinearity issues, thereby affirming the robustness of the model.

**Table 5:** Multicollinearity assessment using VIF values.

**Construct**	**1**	**2**	**3**
1. Psychological distress			
2. Work stress	1.292		1.000
3. WFC	1.292		

VIF values are <3.

### 4.5 Testing the study hypotheses

**Figure 2:**
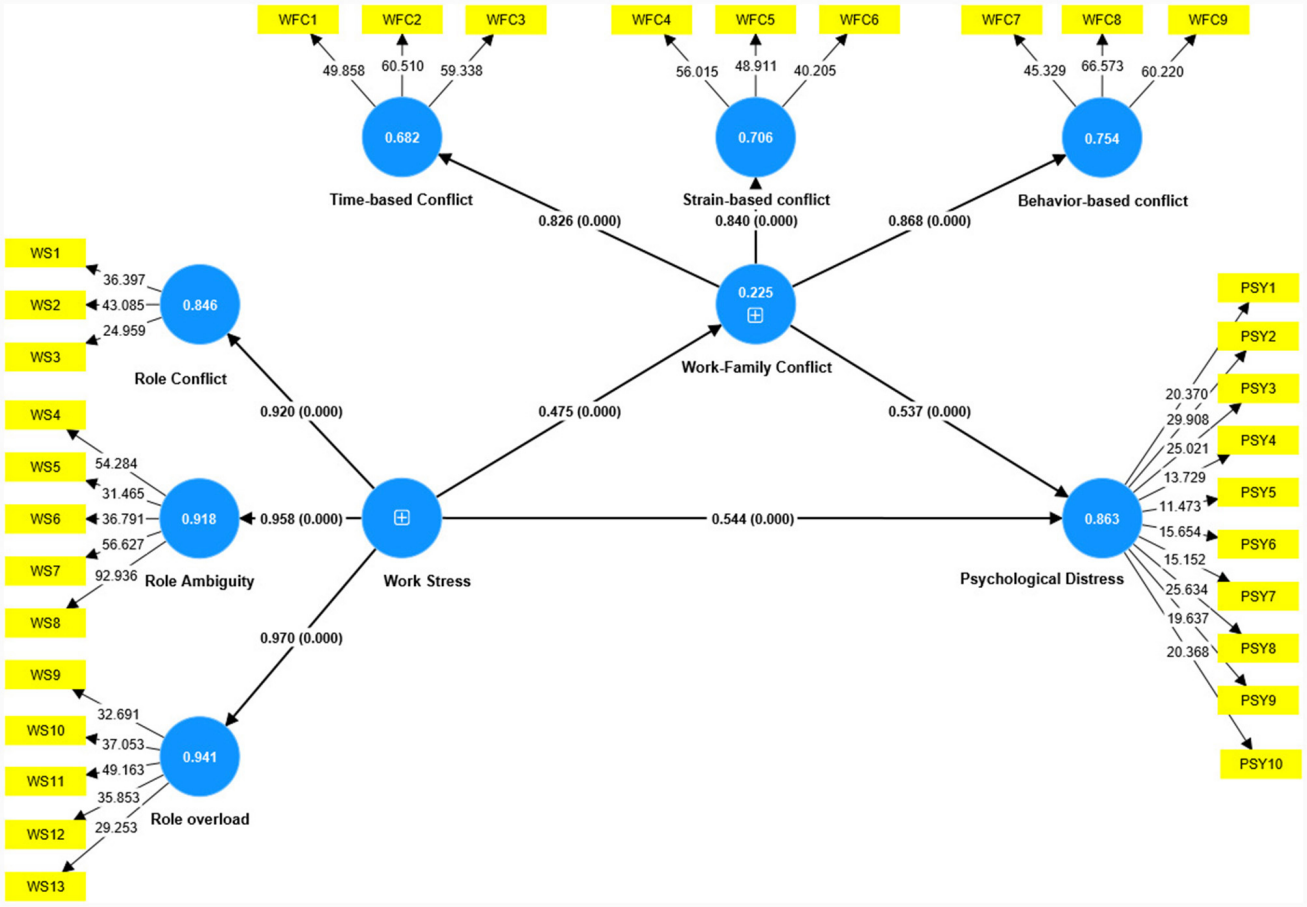
The study's structural model. WS, work stress; WFC, work-family conflict; PSY, psychological distress. The path analysis shows the association between work stress, work-family conflict, and employees' psychological distress. The coefficients presented are standardized linear regression coefficients. Values in the blue circle represent *R*^2^. The value (0.000) means that path coefficients are significant at *p* < 0.001.

This study employed PLS-SEM for hypothesis testing. The assessment of path coefficients was carried out using the bootstrapping technique, specifically involving 5,000 iterations. The research findings derived from the PLS-SEM analysis are presented in Table 6 and Figure 2 for comprehensive examination.

**Table 6:** Structural parameter estimates.

**Hypothesized path**	***B***	***SE***	***t***	**95% CI**		**Result**

				**LL**	**UL**	
WS → PSY	0.544	0.033	16.724^***^	0.482	0.610	Accepted
WS → WFC	0.475	0.045	10.468^***^	0.384	0.563	Accepted
WFC → PSY	0.537	0.030	17.654^***^	0.477	0.594	Accepted
WS → WFC → PSY	0.255	0.027	9.399^***^	0.201	0.308	Accepted

WS, work stress; WFC, work-family conflict; PSY, psychological distress; CI, confidence interval; LL, lower limit; UL, upper limit; WS → PSY, the direct effect of work stress on psychological distress; WS → WFC, the direct effect of work stress on work-family conflict; WFC → PSY, the direct effect of work-family conflict on psychological distress; WS → WFC → PSY, the mediation effect of work-family conflict on the relationship between work stress and psychological distress.

^***^p < 0.001.

The findings presented in Table 6 and depicted in Figure 2 provide insights into the direct influences of work stress on WFC and employees' psychological distress. Additionally, they shed light on the mediating role of WFC in the relationship between work stress and employees' psychological distress. As Hypothesis 1 posits a significant impact of work stress on employees' psychological distress, the empirical results affirm this hypothesis (β = 0.544, *t*-value = 16.724, *P* < 0.001). Furthermore, the empirical analysis reveals that work stress has a substantial and affirmative effect on the perceived WFC (β = 0.475, *t*-value = 10.468, *P* < 0.001). Hence, Hypothesis 2 is corroborated. In alignment with Hypothesis 3, which postulated a significant influence of WFC on employees' psychological distress, the results confirm this hypothesis (β = 0.537, *t*-value = 17.654, *P* < 0.001).

A Bootstrapping technique was employed to ascertain the indirect link between work stress and employees' psychological distress and explore the mediating role of WFC (Elshaer et al., 2023). The findings in Table 5 underscore the significant, positive, and indirect effects of work stress on employees' psychological distress through WFC. Consequently, Hypothesis 4 is validated. In assessing the mediating effect of WFC in this relationship, partial and full mediation models were explored, drawing from the frameworks proposed by Kelloway (1995) and Zhao et al. (2010). These frameworks advocate that full mediation is established when the indirect effects are significant, and the direct effects are not, while partial mediation is indicated when both paths exhibit significance. Based on the results derived from the PLS-SEM analysis, it is evident that WFC partially mediates the relationship between work stress and employees' psychological distress.

## 5 Discussion and implications

### 5.1 Discussion

This study explored the complex interplay between work-related stressors, WFC, and employees' psychological distress in the context of frontline employees in three- and four-star Egyptian resorts. The results provide valuable insights into these variables' direct and mediating effects, shedding light on the intricacies of their relationships. Firstly, the study's findings strongly support Hypothesis 1, indicating that work-related stressors, such as role overload, ambiguity, and conflict, significantly contribute to employees' psychological distress. This finding supports the previous findings (i.e., Iwata et al., 1992; Revicki and Gershon, 1996; Wang and Wang, 2019; Li et al., 2020; and Xiao et al., 2022). This result underscores the detrimental impact of work stress on employees' psychological wellbeing, highlighting the importance of addressing stressors in the workplace to mitigate psychological distress.

Secondly, in the context of work stress-WFC relationship, the findings revealed that work stress has a substantial and positive influence on perceived WFC. This suggests that high levels of work-related stressors can spill over into employees' family lives, creating a conflict between work and family responsibilities. More specifically, the observed substantial and positive influence of work stress on WFC suggests that the pressures and demands experienced in the workplace can spill over into an employee's family life. Work-related stressors such as role overload, ambiguity, and conflict not only affect employees during their working hours but also extend beyond the workplace, creating a conflict between their professional and personal roles. This result aligns with earlier research findings that supported the notion that work stress has a positive and significant impact on WFC (Burke et al., 2013; Ryan et al., 2015; Zhao and Ghiselli, 2016; O'Neill and Follmer, 2020; and Abdou et al., 2022).

Thirdly, the results align with Hypothesis 3, underscoring the significance of WFC as a contributing factor to employees' psychological distress. These findings highlight the adverse effects that conflict between work and family responsibilities can have on employees' psychological wellbeing. The presence of such a relationship is in line with existing research that has consistently demonstrated the detrimental impact of WFC on various aspects of employees' lives, including their psychological distress (Karatepe et al., 2010; Poms et al., 2016; Rubab, 2017; Bilodeau et al., 2020; Barriga Medina et al., 2021; Antino et al., 2022). Based on this finding, it could be concluded that the higher the experience of WFC, the higher the perceived psychological distress. Specifically, when employees face conflicting demands from their work and family roles, it can deplete personal and emotional resources. These resource depletions can result in increased feelings of emotional exhaustion and distress.

Fourthly, regarding the intermediary role of WFC in the relationship between work stress and employees' psychological distress, this study's findings indicate that WFC partially mediates this relationship. In other words, while work stress directly influences psychological distress, part of its impact is directed through its effect on work-family conflict. This finding underscores the importance of recognizing the role of WFC as a pathway through which work stress affects employees' psychological wellbeing. More specifically, in agreement with the previous findings (i.e., Haines III et al., 2008; Shimazu et al., 2010; du Prel and Peter, 2015) and these findings provide substantial support for the notion that adverse conditions and stressors within the workplace have a detrimental impact, subsequently increasing the imbalance between an individual's work and family life. Consequently, this heightened WFC serves as a contributing factor to the increase in psychological distress among employees.

### 5.2 Theoretical implications

The findings of this study carry significant theoretical implications that can be integrated with the job demands-resources (JD-R) model and spillover theory, thereby enriching our comprehension of the underlying dynamics. Firstly, the study's results reinforce the application of the JD-R model in the context of the resort industry, asserting that excessive demands in the workplace, in the form of stressors including role overload, ambiguity, and conflict, can lead to experiencing psychological distress which can manifest as emotional exhaustion, anxiety, and other negative psychological states. Secondly, the study reveals that work stress has a substantial and positive influence on perceived WFC, confirming that work stressors can spill over into employees' family lives. This result resonates with the spillover theory, which suggests that experiences in one domain (work) can impact another domain (family). In this case, high levels of work-related stressors can create a conflict between work and family responsibilities, supporting the idea of negative spillover from work to family. Thirdly, the study emphasized the role of WFC as a contributing factor to employees' psychological distress. This aligns with both the JD-R model and the spillover theory. The JD-R model suggests that WFC can be considered a job demand, as it depletes employees' resources, leading to psychological distress. Spillover theory complements this by highlighting the bidirectional nature of spillover, indicating that the conflict experienced in one domain (in this case, work-to-family) can also negatively affect wellbeing. Fifthly, the findings support the idea that WFC acts as a pathway through which work stress affects employees' wellbeing. Integrating these findings into the JD-R model and spillover theory, we can argue that the negative impact of work-related stressors not only directly affects employees' psychological distress but also indirectly through the escalation of WFC. Finally, the theoretical model developed in this study is a potentially valuable reference for future research in the hospitality industry context, offering insights into both direct and mediated links between job-related stressors and employees' psychological distress.

### 5.3 Practical implications

In addition to theoretical implications, several practical implications arise for resort managers in light of the study's findings. First, resort management should prioritize the management of workplace stressors, particularly role overload, ambiguity, and conflict. Implementing stress reduction programs and offering resources to help employees cope with these stressors can be beneficial in mitigating psychological distress. Second, resort management should provide clear and specific job descriptions and expectations for their employees. This can help reduce role ambiguity and ensure that employees understand their responsibilities, which, in turn, can alleviate psychological distress. Third, employers should assess workloads regularly to prevent excessive role overload. Implementing efficient workload management strategies, such as task prioritization and reasonable goal-setting, can help reduce the strain on employees and enhance their psychological wellbeing. Fourth, resort management can offer employees conflict resolution and interpersonal communication training. Equipping them with the skills to handle conflicts and interpersonal issues effectively can mitigate role conflict and contribute to reduced psychological distress. Fifth, implementing policies and practices that support work-life balance is essential. Encouraging flexible working hours, providing family support programs, and promoting a healthy boundary between work and personal life can help reduce WFC. Sixth, recognizing the significant impact of work-family conflict on psychological distress, resorts can introduce employee wellbeing programs. These programs may include stress management workshops, counseling services, and mental health support to help employees cope with the demands of both work and family roles. Seventh, providing training and awareness programs for employees and supervisors is also vital. These programs can raise awareness about the potential spillover effects of workplace stress into family life and provide strategies to manage and balance these demands effectively. Eighth, resort managers should regularly assess employee wellbeing and levels of psychological pressure they face, whether through follow-up surveys or individual interviews, to provide appropriate psychological and social support and guidance. Finally, resort management should invest in training for supervisors and managers to help them recognize signs of role-related distress in their teams. Training can empower supervisors to provide support and resources when needed.

## 6 Limitations of the study and further research

In the current study, some limitations should be considered. (1) The study concentrated on frontline employees within three- and four-star resorts in Egypt, limiting the applicability and generalizability of the results to different industries, or geographic regions. To better understand the variations in the impact of work-related stressors on psychological distress, future research should explore and compare these findings across diverse industries, job roles, and regions. It is crucial to recognize that cultural, organizational, and societal factors may influence these relationships differently in various geographical locations. Consequently, replicating and extending our findings in varied settings is essential to enhance the external validity of the results. In addition, while our study's specific context is within Egyptian resorts, the implications extend beyond this locale. Organizations in the global hospitality sector, characterized by comparable fast-paced and demanding work environments, may find relevance in our findings. Despite potential differences in stressors and cultural nuances, the overarching themes of addressing work-related stressors, managing work-family conflict, and promoting employee wellbeing are likely to have resonance across diverse borders. (2) The study employed a cross-sectional design, which provides a snapshot of the relationships at one point in time. Longitudinal or experimental designs offer insights into the dynamics of work-related stressors, work-family conflict, and psychological distress over time. (3) This study primarily focused on the mediating role of WFC. Further research could investigate other potential mediating variables to provide a more comprehensive view of the relationships. The effectiveness of interventions, such as stress management programs or work-family balance initiatives, in reducing psychological distress among employees facing work-related stressors should be explored. (4) Data collection relied on self-report measures, which may introduce response bias and subjectivity. Future research could consider combining quantitative data with qualitative research methods to gain deeper insights into employees' experiences in the context of work-related stressors and psychological distress. (5) This study was mainly built on the job demands-resources (JD-R) model and the spillover theory in the link between WS, WFC, and psychological wellbeing. Further studies may incorporate another theory, such as boundary theory, to enrich the understanding of the link between these variables. Boundary theory searches for boundaries between different life domains, mainly focusing on the work-family interface. It explores how individuals manage the lines between these domains, influencing their wellbeing across various aspects of life. Boundary permeability, flexibility, and segmentation may be included. (6) We focus in this study on work stress as a higher-order construct encompassing three sub-factors: role overload, ambiguity, and conflict. While this hierarchical model provides valuable insights into the overall impact of work stress, it does not allow us to analyze the specific effects of each individual sub-factor on psychological wellbeing. As a limitation, we acknowledge that we cannot isolate the unique contributions of each work stress dimension within this framework. In addition, future research employs a three-factor model of work-family conflict (WFC) as a mediator. This model, encompassing time-based, strain-based, and behavior-based conflict as distinct dimensions, could offer more valuable insights into how specific aspects of work stress impact psychological wellbeing through different facets of WFC.

## Data availability statement

The original contributions presented in the study are included in the article/Supplementary material, further inquiries can be directed to the corresponding author.

## Ethics statement

The studies involving humans were approved by the Deanship of the Scientific Research Ethical Committee, King Faisal University. The studies were conducted in accordance with the local legislation and institutional requirements. The participants provided their written informed consent to participate in this study.

## Author contributions

AHB: Conceptualization, Data curation, Formal analysis, Funding acquisition, Investigation, Methodology, Project administration, Resources, Software, Supervision, Validation, Visualization, Writing—original draft, Writing—review & editing. ME-A: Data curation, Formal analysis, Funding acquisition, Methodology, Validation, Writing—original draft. EM: Data curation, Formal analysis, Funding acquisition, Investigation, Methodology, Writing—original draft. HA: Formal analysis, Investigation, Methodology, Resources, Software, Writing—original draft. AR: Data curation, Formal analysis, Investigation, Resources, Software, Validation, Writing—review & editing. MYA: Data curation, Formal analysis, Methodology, Visualization, Writing—review & editing. ASMA: Data curation, Funding acquisition, Investigation, Methodology, Validation, Writing—review & editing. AMA: Data curation, Formal analysis, Funding acquisition, Methodology, Validation, Writing—original draft. MOA: Formal analysis, Methodology, Software, Visualization, Writing—original draft. JA: Formal analysis, Funding acquisition, Methodology, Validation, Writing—review & editing. MM: Data curation, Formal analysis, Investigation, Methodology, Visualization, Writing—review & editing. AE: Writing—review & editing, Supervision, Project administration, Resources. SA: Formal analysis, Funding acquisition, Investigation, Methodology, Resources, Writing—review & editing.
