# Supplementary material for: Work stress, work-family conflict, and psychological distress among resort employees: a JD-R model and spillover theory perspectives
Source: Front Psychol. 2024 Feb 14;15:1326181. doi: 10.3389/fpsyg.2024.1326181 (PMC10899679; doi:10.3389/fpsyg.2024.1326181)
Supplement: Supplementary file 1 [file Data_Sheet_1.PDF]

Supplementary Materials

Study's constructs and their related items

Construct	Items	Statement
**Work stress**		
Role conflict	WS1	I often get involved in situations in which there are conflicting requirements.
	WS2	I receive incompatible requests from two or more people.
	WS3	I have to do things that should be done differently under different conditions.
Role ambiguity	WS4	I do not have clear planned goals and objectives for my job.
	WS5	I do not know exactly what is expected of me.
	WS6	I do not know what my responsibilities are.
	WS7	I do not feel certain about how much responsibility I have.
	WS8	My responsibilities are not clearly defined.
Role overload	WS9	There is a need to reduce some parts of my role.
	WS10	I feel overburdened in my role.
	WS11	I have been given too much responsibility.
	WS12	My workload is too heavy.
	WS13	The amount of work I have to do interferes with the quality I want to maintain.
**Work-family conflict**		
Time-based conflict	WFC1	working in resorts keeps employees from their family activities more than they should be.
	WFC2	The long working hours/periods keep employees from participating equally in household responsibilities and activities.
	WFC3	Employees have to miss their family activities due to the amount of time they should spend on work.
Strain-based conflict	WFC4	Working in resorts is a frazzle to participate in family activities/responsibilities.
	WFC5	Employees are so emotionally drained when they get home from work that it prevents them from contributing to their families.
	WFC6	Due to the work pressures, employees transfer their strain to their family
Behavior-based conflict	WFC7	The problem-solving behaviors used in the job are not effective in resolving problems at home.
	WFC8	The commitment at work (i.e., shifting the work periods) would be counterproductive at home.
	WFC9	Behavior that is effective and necessary for work does not help the employees to be better parents and spouses.
**Psychological distress: In the past 30 days, how often**		
Psychological distress	PSY1	did you feel tired out for no good reason?
	PSY2	did you feel nervous?
	PSY3	did you feel so nervous that nothing could calm you down?
	PSY4	did you feel hopeless?
	PSY5	did you feel restless or fidgety?
	PSY6	did you feel so restless that you could not sit still?
	PSY7	did you feel depressed?
	PSY8	did you feel that everything was an effort?
	PSY9	did you feel so sad that nothing could cheer you up?
	PSY10	did you feel worthless?
